# Reply to Strømmen, M.; Skråstad, R.B. Comment on “Campuzano-Donoso et al. Alcohol and Substance Use After Bariatric Surgery: Nutritional Risks and Clinical Implications in Long-Term Postoperative Care. *Nutrients* 2026, *18*, 932”

**DOI:** 10.3390/nu18213534

**Published:** 2026-10-27

**Authors:** Martín Campuzano-Donoso, Claudia Reytor-González, Gerardo Sarno, Martha Montalvan, Luigi Barrea, Giovanna Muscogiuri, Ludovica Verde, Giuseppe Annunziata, Daniel Simancas-Racines

**Affiliations:** 1 Center for Evidence Ecosystems, Implementation Science, and Decision-Making (CIDES), Universidad Tecnológica Indoamérica, Ambato 180150, Ecuador; martincd01@hotmail.com (M.C.-D.); claudiareytor@gmail.com (C.R.-G.); 2 San Giovanni di Dio e Ruggi D’Aragona University Hospital, Scuola Medica Salernitana, 84131 Salerno, Italy; gsarno79@yahoo.it; 3 Escuela de Medicina, Universidad Espíritu Santo, Samborondón 0901952, Ecuador; mmontalvanmd53@gmail.com; 4 Department of Psychology and Health Sciences, Università Telematica Pegaso, Centro Direzionale F2, 80143 Naples, Italy; luigi.barrea@unipegaso.it; 5 Centro Italiano per la Cura e il Benessere del Paziente con Obesità (C.I.B.O.), Unità di Endocrinologia, Diabetologia e Andrologia, Dipartimento di Medicina Clinica e Chirurgia, Università degli Studi di Napoli Federico II, 80131 Naples, Italy; giovanna.muscogiuri@gmail.com (G.M.);; 6 Division of Endocrinology, Department of Medicine, The University of Arizona College of Medicine, Tucson, AZ 85724, USA; 7 Dipartimento di Medicina Clinica e Chirurgia, Unità di Endocrinologia, Diabetologia ed Andrologia, Università degli Studi di Napoli Federico II, 80131 Naples, Italy; 8 Cattedra Unesco “Educazione Alla Salute E Allo Sviluppo Sostenibile”, Università degli Studi di Napoli Federico II, 80131 Naples, Italy; 9 Department for the Promotion of Human Sciences and Quality of Life, San Raffaele Open University, 00166 Rome, Italy; giuseppe.annunziata@uniroma5.it

We thank the authors for their Comment [1] on our Review [2]. They raise three related concerns: whether describing the Alcohol Use Disorders Identification Test–Consumption (AUDIT-C) as a validated instrument implies validation specifically in bariatric populations; whether postoperative changes in ethanol pharmacokinetics limit the interpretation of conventional screening scores; and whether phosphatidylethanol (PEth) could complement self-reported alcohol consumption. We address these points in turn.

Regarding validation, we agree that the wording in our review could have been more precise. The expressions “validated screening (e.g., AUDIT-C)” in the Abstract and “standardized, validated screening instruments” in Section 6.2 referred to validation in general clinical settings, not specifically after metabolic and bariatric surgery (MBS). AUDIT-C has been validated as a brief screening instrument for unhealthy drinking in general medical populations [3], but bariatric-specific sensitivity, specificity, predictive values, and optimal thresholds have not been established. We acknowledge that our original wording did not make this distinction sufficiently clear.

This limitation does not, however, render AUDIT-C uninformative in postoperative care. Maciejewski et al. [4] used repeated AUDIT-C assessments in 2608 US veterans undergoing sleeve gastrectomy or Roux-en-Y gastric bypass and identified increases in unhealthy alcohol use during long-term follow-up. This demonstrates its utility for describing longitudinal changes in reported drinking, but it does not validate conventional AUDIT-C cutoffs as estimates of postoperative physiological risk. The distinction is between measuring consumption patterns and inferring the consequences of that consumption.

Concerning ethanol pharmacokinetics, we agree with the commenters that the same reported alcohol intake can produce greater systemic exposure after surgery. Controlled alcohol-administration studies have shown faster absorption, shorter times to peak concentration, and higher peak blood alcohol concentrations after Roux-en-Y gastric bypass and sleeve gastrectomy [5,6]. The prospective BAR-TRIAL further demonstrated persistent changes in peak concentration, time to peak, and total exposure over three years [7]. These findings do not alter what the three AUDIT-C items ask, but they do mean that conventional consumption thresholds should not be interpreted as thresholds for safe drinking after MBS.

The commenters also caution that AUDIT-C may underestimate risk or yield false-negative results. We share the clinical concern: a low or unchanged score cannot, by itself, exclude alcohol-related harm when physiological exposure per drink has increased. Nevertheless, a false-negative screening result, in the diagnostic-accuracy sense, must be defined against a specified target condition and reference standard. Available evidence has not established that surgery-related changes in ethanol pharmacokinetics reduce AUDIT-C sensitivity for detecting unhealthy drinking or alcohol use disorder. The better-supported concern is therefore false clinical reassurance when a consumption score is treated as a measure of postoperative intoxication or harm.

Turning to the proposal to use PEth, we agree that this biomarker can provide useful information alongside self-report. In a preoperative bariatric sample, Miller-Matero et al. [8] found that 8 of 13 participants (61.5%) with PEth concentrations indicating moderate or heavy alcohol use did not have an elevated AUDIT-C score. This discordance is clinically relevant, but the small, preoperative comparison does not establish postoperative diagnostic accuracy or demonstrate that altered ethanol pharmacokinetics caused the discordance. More recently, Berggren et al. [9] measured PEth longitudinally in 1238 patients and observed an increase in the proportion exceeding a high-consumption threshold after surgery, particularly among men. That study supports the potential value of PEth for follow-up, but it did not validate PEth against AUDIT-C or a structured diagnosis of alcohol use disorder.

PEth and AUDIT-C are thus complementary rather than interchangeable. AUDIT-C documents reported drinking frequency, quantity, and episodic heavy consumption, whereas PEth provides a direct biomarker of recent alcohol exposure. PEth cannot, on its own, diagnose alcohol use disorder, reconstruct the timing of individual drinking episodes, or quantify peak blood alcohol concentration after a particular drink. Its formation and elimination also vary between individuals, and bariatric-specific interpretive thresholds have not been established [10,11]. We therefore support its selective use as an adjunct to clinical assessment, not as a substitute for questioning patients about alcohol use and its consequences.

In practical terms, our recommendation is to retain AUDIT-C as an accessible initial screen while interpreting its results in the broader clinical context. Relevant considerations include procedure type, time since surgery, previous alcohol or substance use, psychiatric history, symptoms, and evidence of nutritional or hepatic complications. A positive screen, concerning symptoms, or discrepancy between reported intake and clinical findings should prompt further assessment using established diagnostic criteria, with the full AUDIT and PEth testing considered when they could inform management. Neither AUDIT-C nor PEth alone should be used to characterize overall alcohol-related risk after MBS.

Finally, we agree that the appropriate next step is bariatric-specific validation rather than adoption of untested screening thresholds. Prospective studies should compare AUDIT-C and the full AUDIT with structured diagnostic interviews and clinically meaningful alcohol-related outcomes, examine whether performance differs by procedure and postoperative interval, and determine whether adding PEth improves risk classification. We thank the commenters for identifying a limitation in how our original recommendation was phrased and for helping to clarify the distinct roles of self-report, biomarkers, and clinical judgment in long-term postoperative care.

## Data Availability

No new data were created or analyzed in this Reply. Data sharing is not applicable.
